# Compressibility of Confined Fluids from Volume Fluctuations

**DOI:** 10.1021/acs.langmuir.5c03971

**Published:** 2025-12-15

**Authors:** Jason Ogbebor, Santiago A. Flores Roman, Geordy Jomon, Gennady Y. Gor

**Affiliations:** † Department of Materials Science and Engineering, 553651 Massachusetts Institute of Technology, Cambridge, Massachusetts 02139, United States; ‡ Otto H. York Department of Chemical and Materials Engineering, 122389 New Jersey Institute of Technology, Newark, New Jersey 07102, United States

## Abstract

When fluids are confined in nanopores, many of their properties deviate from bulk. These include bulk modulus, or compressibility, which contributes to the mechanical properties of fluid-saturated porous solids. Such properties are of importance for exploration and recovery of coal-bed methane and shale gas. We developed a new molecular simulation approach for calculating compressibility of confined fluids, and applied it to methane in carbon nanopores. The method is based on volume fluctuations in the isothermal–isobaric ensemble, made possible through integrated potentials. Our method is more than an order of magnitude faster than the Monte Carlo approach, and allows calculations for pore sizes up to 100 nm. Our simulations predicted an increase in the fluid bulk modulus by a factor of 4 in 3 nm slit pores, and showed a gradual decrease with the increase of the pore size, so that at 100 nm, the deviation from the bulk is less than 5%.

## Graphical abstract

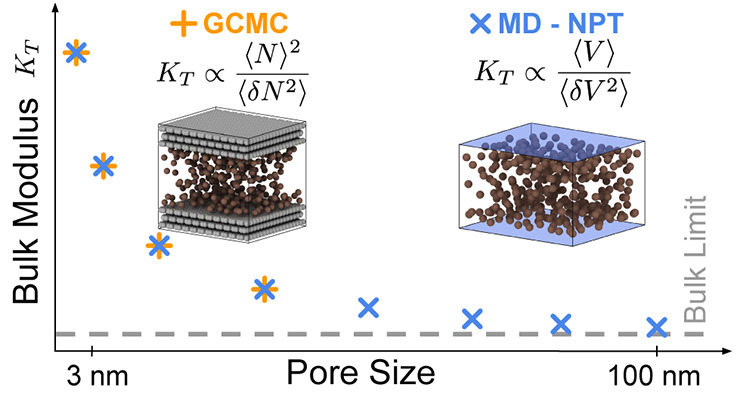

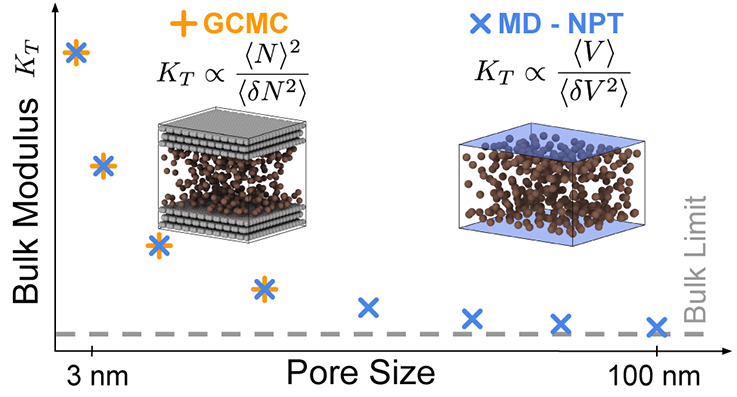

## Introduction

The phenomenon of changing fluid properties under nanoconfinement has been studied by a number of works which have demonstrated significant alteration in density, phase transition temperatures, diffusivity, and other fluid properties.^1–3^ The magnitude of these effects depends on the pore size and the strength of solid–fluid interactions.^3^ Compressibility or bulk modulus is one of the properties altered by confinement.^4^ Bulk moduli of both solid and fluid components determine the poroelastic behavior of fluid-saturated rocks, relevant to the exploration and extraction of hydrocarbons.^5^

Ultrasound propagation experiments on fluid-saturated nanoporous media have shown significant deviation in the bulk modulus of several fluids under confinement.^6–13^ It has also been confirmed by grand canonical Monte Carlo (GCMC) simulations of argon, nitrogen, and methane in pores of various shapes. These studies showed that for the pores in the range of sizes 2–10 nm, the bulk modulus shows a linear dependence on the inverse of the pore diameter.^14–16^ This mirrors the effect of confinement on the freezing point given by the Gibbs–Thomson equation,^3,17^ but the observed trend did not intersect with the modulus of the bulk fluid at the limit of an infinitely large pore, suggesting that the true dependence should eventually plateau with increasing pore size. Further, characterization of shale formations has shown that fluid-saturated pore sizes vary widely from ∼0.3 nm to 100 μm,^18–20^ so properties of hydrocarbons are of interest for a wide range of pore sizes.

While calculations of bulk modulus of confined fluids using GCMC are straightforward, the computational cost becomes prohibitive for large pore sizes. This limitation motivates the development of alternative simulation methods to investigate the effects of confinement on the bulk modulus of fluids in a pore of ever increasing size. In this article, we introduce a method for calculating the fluid bulk modulus using a well-established relation from statistical mechanics which previously could only be applied to bulk fluids, and for which the speed of the calculation scales well with the size of the pore.

To put this new approach into context, it is appropriate to briefly take account of the current established methods and identify their limitations in modeling confined fluid compressibility in large pores. The isothermal compressibility β_*T*_, and its reciprocal, isothermal bulk modulus *K*_*T*_, are defined as

1 $$K_{T}=\beta_{T}^{-1}=-V{\left(\frac{\partial P}{\partial V}\right)}_{T}$$

where *V* is the volume of the system, *P* is the fluid pressure, and the temperature *T* is held constant. This macroscopic definition of bulk modulus assumes that the system pressure is isotropic.

Through statistical mechanics, derivative thermodynamic properties of a system can be calculated from the fluctuation of extensive variables in a given ensemble. In the grand canonical ensemble, *V*, *T*, and chemical potential μ are prescribed and held constant, while the number of particles *N* in the system is allowed to vary, most commonly by Monte Carlo (MC) moves. The bulk modulus of such a system can be calculated through the expression

2 $$K_{T}=\frac{k_{B}T\langle N\rangle^{2}}{V\left\langle \delta N^{2}\right\rangle }$$

where *k*_B_ is the Boltzmann constant, angled brackets represent an ensemble average, and ⟨*δN* ^2^⟩ = ⟨*N* ^2^⟩ – ⟨*N*⟩^2^ is the variance of *N*. In the canonical (*NVT*) ensemble, *N*, *V*, and *T* are held constant. This leaves fluctuations in the internal virial W and hypervirial function X , from which the bulk modulus can also be calculated as

3 $$K_{T}=\frac{1}{V}\left(Nk_{B}T+\left\langle W\right\rangle +\left\langle X\right\rangle -\frac{\left\langle \delta W^{2}\right\rangle }{k_{B}T}\right)$$

in which $\left\langle \delta W^{2}\right\rangle$ is the variance of the virial, defined similarly to the variance of *N* in eq 2.^21^

These methods of modeling bulk modulus, while effective for simulations of simple fluids confined in a relatively small pore, are not easily applied to all fluids or to a large pore. In GCMC simulations of dense fluids, it is difficult to reach and sample an equilibrated system in a reasonable amount of time because particle insertion becomes progressively inefficient as the size of the system increases. This approach is further hampered by the introduction of Coulombic and many-body interactions. In contrast, molecular dynamics (MD) simulations of simple fluids in the *NVT* ensemble are relatively fast, but the calculations required to apply the virial fluctuation method add further computational cost. Additionally, it is not applicable for molecules with flexible angles and dihedrals.

A method which has neither of the aforementioned limitations can be applied in the isothermal–isobaric (*NPT*) ensemble, in which *N*, *P*, and *T* are held constant, while the volume of the system fluctuates. In such a system, the bulk modulus can be calculated as

4 $$K_{T}=\frac{k_{B}T\left\langle V\right\rangle }{\left\langle \delta V^{2}\right\rangle }$$

where ⟨*δV* ^2^⟩ is the variance of *V*, defined similarly to the variance of *N* in eq. 2. This method is routinely applied to bulk fluids and solids. However, the application of eq 4 to fluids confined by solid pore walls is complicated by the following challenges. (1) Isotropic volume fluctuations will change the pore size, and thus the bulk modulus of the fluid during the course of the simulation. (2) Even if volume fluctuations are constrained so that the pore size is constant, it would require the real-time addition and removal of atoms making up the solid walls to keep the fluid confined along the now fluctuating edges of the pore volume. (3) Although the atoms comprising the solid walls can be “frozen” and do not contribute to barostat calculations, the total volume of the simulation box (and thus the variance of that quantity) will include the contribution of the pore walls, which must be removed. To access the performance benefits of using eq 4, the method proposed herein avoids the aforementioned issues by using infinite integrated potentials to represent the confining walls and allowing the volume to fluctuate only in the remaining periodic dimensions. This study demonstrates that eq 4 is valid even when volume fluctuations are anisotropic. We also confirm that the spatial-average bulk modulus of a fluid under nanoscale confinement can be obtained through volume fluctuations, a method which is traditionally applied solely to isotropic fluids.

## Methods

### Integrated Potentials

Integrated potentials are commonly used in MC and MD simulations to modify the potential landscape experienced by atoms in the system, primarily to save computational time.^22,23^ For example, a flat wall of frozen atoms interacting with a fluid can be replaced by a single immaterial plane which imposes a force on a fluid particle as a function of that particle’s distance from the plane. This potential has approximately the same effect on the fluid particles as would a physical wall without calculating the interaction potential contributed by every wall atom. They are especially useful in classical density functional theory (cDFT) calculations.^24^

To determine the most accurate form of the potential, we first account for the contribution of each wall atom. We use as a starting point the pairwise 12-6 Lennard-Jones (LJ) interaction potential commonly used in molecular simulations of nonpolar fluids, which quantitatively reproduces the bulk modulus of methane:^16^

5 $$U=4\epsilon \left[{\left(\frac{\sigma }{r}\right)}^{12}-{\left(\frac{\sigma }{r}\right)}^{6}\right]$$

Here *U* is the potential energy applied to the two interacting particles, ϵ and σ are respectively the energy and distance parameters characterizing the interaction, and *r* is the distance between the two particles. Interactions are normally truncated at some distance *r*_cut_ so that the potential between particles further than this distance is set to zero. Typically, pore walls are represented by a number of stationary atoms, each of which interacts with fluid particles according to eq 5, and are arranged in the desired pore geometry. In this work, slit pores are represented by two infinite planes between which the fluid is confined, and cylindrical pores are represented by an infinite cylindrical shell (see Figure 1).

**Figure 1:**
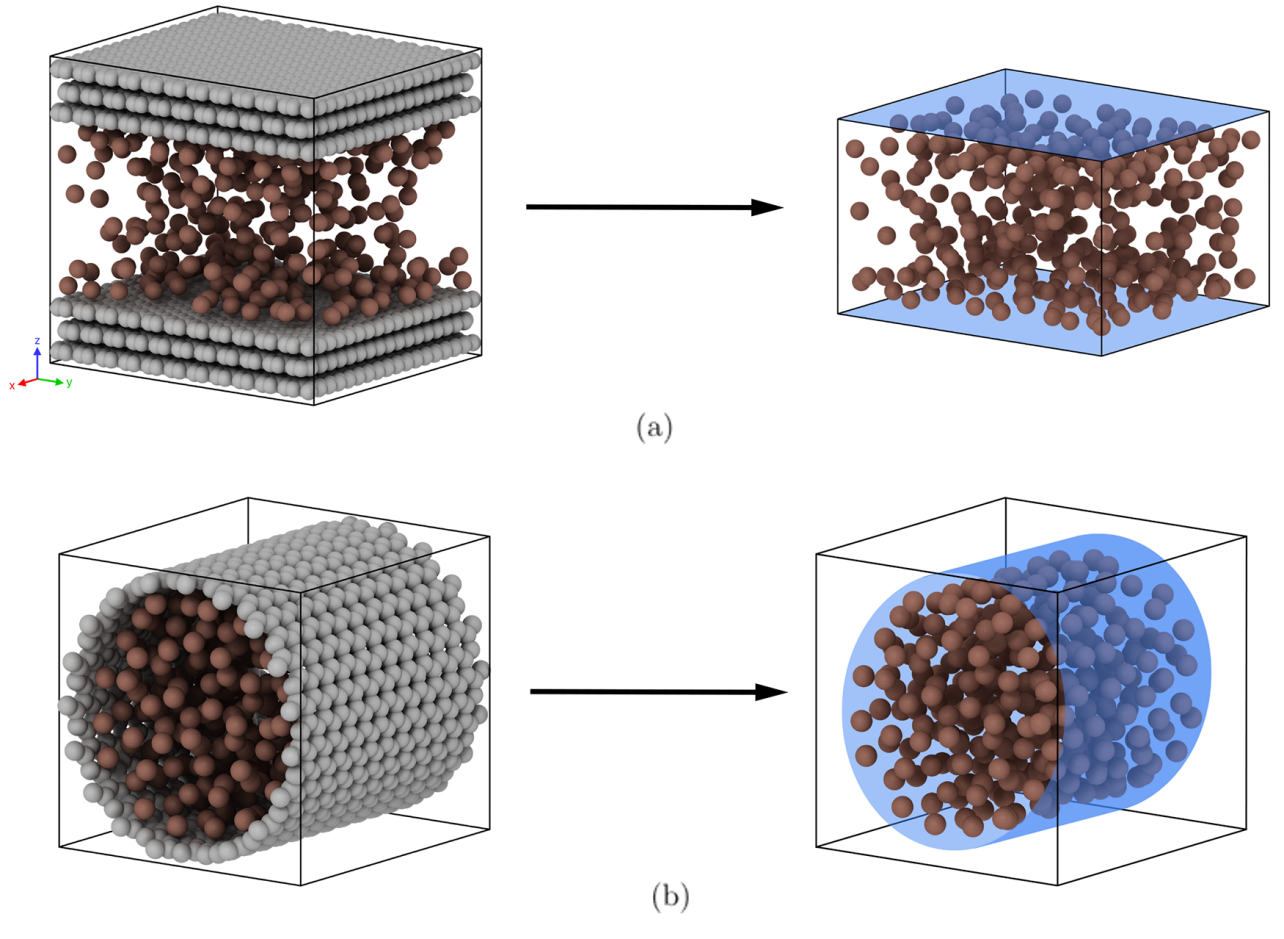
Schematic simulation snapshots of methane confined in 3 nm (a) slit and (b) cylindrical pores with atomistic walls (left) and integrated potential walls (right).

The potential energy landscape experienced by a fluid atom due to flat walls is obtained by twice-integrating eq 5 along the two dimensions with which the wall is parallel, yielding^22^

6 $$U_{\mathrm{sf}}=\overset{n}{\underset{i=1}{\sum }}\left[4\pi \rho_{s}\epsilon_{\mathrm{sf}}\sigma_{\mathrm{sf}}^{2}\left(\frac{1}{5}{\left[\frac{\sigma_{\mathrm{sf}}}{r_{i}}\right]}^{10}-\frac{1}{2}{\left[\frac{\sigma_{\mathrm{sf}}}{r_{i}}\right]}^{4}\right)\right]$$

where ρ_s_ is the effective surface density of each wall layer, the subscript “sf” denotes interaction parameters as in eq 5 between the solid and fluid atoms, *n* is the total number of layers in both walls (such as graphene layers in graphitic walls), and *r*_*i*_ is the distance between a fluid atom and the *i*th wall layer.

The potential energy landscape experienced by a fluid atom confined in a cylindrical pore is obtained by integrating eq 5 in cylindrical coordinates, yielding the potential developed by Tjatjopoulos et al.^25^

7 $$U_{\mathrm{sf}}\left(r,R\right)=2\pi \rho_{s}\sigma_{\mathrm{sf}}^{2}\epsilon_{\mathrm{sf}}\left[\psi_{6}\left(r,R,\sigma_{\mathrm{sf}}\right)-\psi_{3}\left(r,R,\sigma_{\mathrm{sf}}\right)\right]$$

where *r* is the distance between the fluid atom and the central axis parallel to the cylinder, *R* is the cylinder radius, and ψ_*n*_ is a function defined in eq S1 of the Supporting Information. For more comprehensive details the reader is directed to refs. 22,25

### LAMMPS Implementation

We implemented this method for both slit and cylindrical pores in LAMMPS (Large Atomic and Molecular Massively Parallel Simulator),^26^ which has built-in support for integrated potentials. Slit pore walls were created using a custom command similar to fix wall/lj1043, but modified to impose the potential defined in eq 6. Two planes were defined to create the slit pore: one at both ends of the simulation box in the *z*-direction. Different pore sizes were modeled by modifying the height of the simulation box. To test that eq 6 is an accurate representation of slit pore confinement, we compared the fluid density profiles in a 3 nm slit pore using both atomistic walls and the integrated potential, included in the Supporting Information (Figure S1).

Modeling cylindrical pores in LAMMPS first required the creation of the cylindrical region onto which the wall potential would be mapped. None of the wall potentials currently available in LAMMPS adequately model the landscape described by eq 7. To remedy this, it was necessary to write the potential explicitly as an additional option. The Tjatjopoulos potential was then applied to this region using the fix wall/region command. For the full syntax used to create slit and cylindrical pore walls, see the Supporting Information.

Initial configurations were generated by assigning random positions of methane molecules in the pore. Then, the LAMMPS default energy minimization technique was applied to avoid any molecule–molecule or molecule–wall overlap. To determine the number of molecules to be inserted, we reproduced adsorption of methane in cylindrical and slit pores using GCMC simulations (from 3 to 10 nm in size) and fitted the resulting excess adsorbed amount, *N*_ex_ = (ρ_f_ – ρ_b_)*V*_f_, according to the Gamma distribution

8 $$N_{\mathrm{ex}}\left(V_{f};B,\alpha ,\theta \right)=B\frac{V_{f}^{\alpha -1}e^{-V_{f}/\theta }}{\theta^{\alpha }\Gamma \left(\alpha \right)}$$

where *B*, α, and θ are the fitting parameters, and *V*_f_ is the volume of the fluid in the pore (*V*_f_ = *H*_int_ × *A* for slits and *V*_f_ = π­[*H*_int_^2^/4] × 3*r*_cut_ for cylinders). *H*_int_ = *H* – 1.7168σ_sf_ + σ_ff_ is the internal pore size and *A* is the area of a pore wall.^14^ *r*_cut_ is the cutoff radius of solid–fluid interactions. The shape parameter, α, is adjusted according to the pore-shape (α ≈ 1.5 for cylindrical pores and α ≈ 1 for slit pores), while the scale and magnitude parameters, θ and *B*, are dependent on the thermodynamic conditions. Extrapolating the adsorbed amount of methane allows us to avoid running lengthy GCMC simulations of large systems (pores larger than 10 nm in this work) and save significant computational time. Validation of eq 8 can be found in the Supporting Information.

The resulting adsorbed amount is

9 $$N=\rho_{b}V_{f}+N_{\mathrm{ex}}$$

where ρ_b_ is the density of bulk methane, and *N*_ex_ comes from eq 8. *N*_ex_ obtained from the Gamma distribution is strictly positive, and decreases with increasing pore size. For sufficiently large pores, the dominant term will be ρ_b_ *V*_f_. Therefore, the density of confined methane will approach the density of bulk methane.

To reduce any possible finite-size effects in volume oscillations, we set initial wall areas of *A* = *H* × *H* for slit pores and *A* = *πH* × 3*r*_cut_ for cylindrical pores. Figure S4 in the Supporting Information shows that increasing *A* even further would show a negligible improvement of *K*_*T*_ predictions from MD simulations.

**Figure 2:**
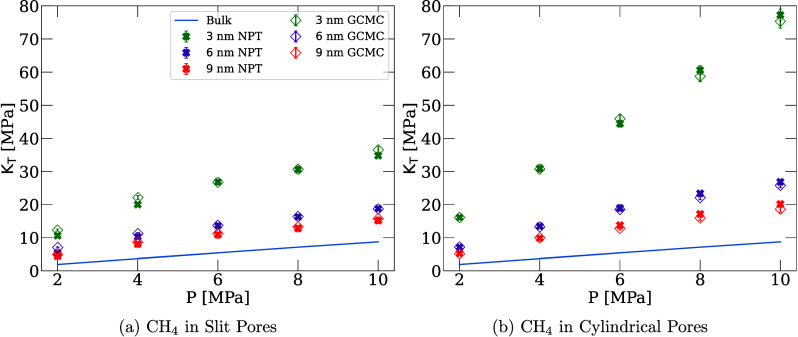
Bulk modulus of supercritical methane as a function of reservoir pressure in (a) carbon slit and (b) cylindrical pores. The filled “×” markers are the results calculated using the new method, from eq 4. The empty markers represent results calculated from eq 2 (diamonds). Error bars represent the standard deviation calculated using the bootstrap method.^32^ The solid line is the modulus of bulk methane calculated from MD simulations using eq 4. In (a), the GCMC data are taken from ref 16. In (b), the GCMC data were calculated in this work. To relate the reservoir pressure to the chemical potential of methane, we used the CoolProp Python library.^33^

MD simulations in slit pores began with equilibration in the canonical ensemble for 1 × 10^6^ timesteps (2 × 10^6^ in cylinders) with one time step set to 0.1 fs. During this phase, the normal components of the system’s pressure tensor (*P*_*xx*_, *P*_*yy*_, *P*_*zz*_) were averaged using the compute pressure and fix ave/time commands. The ensemble was then switched from *NVT* to *NPT*, in which the calculated pressure values are passed to the Nosé-Hoover barostat and volume fluctuations are measured for 2 × 10^7^ timesteps.

For slit pores, only the pressure in the x- and *y*-directions (those directions which are parallel to the pore walls) are specified in the fix npt command with the aniso option for the barostat, thus allowing the volume of the fluid to fluctuate without changing the pore size. Both the x- and y-components specified in the fix npt command use the same value: the arithmetic mean between ⟨*P*_*xx*_⟩ and ⟨*P*_*yy*_⟩. In cylindrical pores, only ⟨*P*_*xx*_⟩ is given to the barostat, thus allowing fluctuations only in the *x*-direction.

Care must be taken not to confuse the pressure of the bulk reservoir with the pressure of the confined fluid, or to assume that these quantities are equivalent.^27,28^ The normal components of the pressure tensor had to be measured over the course of the *NVT* simulation because the pressure in the pore is known to differ from that of the bulk reservoir with which the pore fluid is in equilibrium (i.e., it would be physically inaccurate to apply a pressure of 10 MPa to methane confined in a 3 nm pore based on the assumption that the fluid pressure is equal to the reservoir pressure). As pore size increases toward infinity, the pressures in the periodic dimensions should gradually approach the pressure of the reservoir, and we observed this in our simulations.

Over the course of the *NPT* simulation, volume was collected once every 10^3^ timesteps, and the first 5 × 10^6^ steps were skipped to allow volume to equilibrate before calculating the bulk modulus through eq 4. In both slit and cylindrical pores, the volume used was the fluid-accessible pore volume (*V*_f_ in eq 8). Slit pores used 3 graphene layers in each wall (*n* = 3 in eq 6), and cylindrical pores were single-layered.

The choice of a smaller-than-normal time step was made to reduce the volatility of volume fluctuations and increase the accuracy of the dynamics integration. The long simulation time for equilibration and production of statistical data accommodates larger pores, in which dynamics are somewhat slower than in small pores. The criterion for judging the statistical quality of the collected volume data was the Gaussian shape of the resulting histograms, indicative of a well-sampled ensemble. Shorter simulation times are possible, so long as one is careful to check the state of equilibration, collect a data set of sufficient size, and avoid correlated data points. A brief discussion of the Gaussian shape statistical criterion is provided in the Supporting Information.

All interaction parameters can be found in Table S1 of the Supporting Information. One must be careful in the choice of the *r*_cut_ parameter for solid–fluid interactions, which has a sensitive effect on the structure and dynamics of the fluid close to the walls. It is this region which is primarily responsible for the increased bulk modulus of confined fluids. In this work, the value of *r*_cut_ between the integrated potential and fluid atoms was set equal to the cutoff distance between fluid atoms, *r*_cut_ = 1.2 nm. This is the same value used between carbon atoms (in the wall) and united-atom methane in previous simulation studies of confined methane.^16^ We confirm that the effects of confinement in the slit pores is effectively identical between the solid walls and integrated potentials by comparing density profiles in the Supporting Information (see Figure S1).

## Results

### Methane in Carbon Slit and Cylindrical Pores

To demonstrate the proposed method, we chose to simulate the system of supercritical methane (represented by a united-atom model^29^) confined in graphitic slit pores, which has practical importance as a proxy for modeling shale gas or coal bed methane.^30^ This same system was studied by Corrente et al.,^16^ in which the authors used both eq 2 and eq 3 to calculate the bulk modulus of the confined fluid. Calculations were carried out for pore sizes ranging from 3 to 9 nm and reservoir pressures from 2 to 10 MPa. It is worth noting that the bulk modulus as calculated by eq 4 is sensitive to the exact number of fluid atoms in the pore, which was determined by GCMC simulations. To extend this method to larger pore sizes without running lengthy GCMC simulations, it was necessary to extrapolate the number of atoms as described earlier in this article. Furthermore, calculating the bulk modulus from eq 4 for larger pores may introduce some finite-size effects. An analysis of these effects on the modulus is given in the Supporting Information.

The calculated bulk moduli are shown in Figure 2a, compared with GCMC results. While sorption-ultrasonic experiments can provide data on moduli of fluids in nanopores,^4,7,11,13,31^ to our knowledge there is no experimental literature data on the bulk moduli of confined methane. Across all pore sizes and reservoir pressures, we observed quantitative agreement between eq 2 and the method introduced in this article. This comparison confirms the applicability of eq 4 to nanoconfined fluids. Details regarding the calculation of statistical errors can be found in the Supporting Information.

Having demonstrated that our method is effective for slit pores, we extend it to another geometry by testing the same solid–fluid pair but in cylindrical pores. As with slit pores, GCMC simulations were employed to calculate the mean density of adsorbed fluid at equilibrium, which was then used to generate the initial configurations for MD simulations in LAMMPS. eqs 6 and 7 both used solid–fluid interaction parameters calculated via conventional Lorentz–Berthelot mixing rules.^34^

The bulk moduli as computed through eqs 2 and 4 are shown in Figure 2b in pore sizes mirroring the results for slits. As with slit pores, agreement is observed between the bulk moduli calculated from both methods. For both pore shapes, the modulus shows a monotonic increase with the pressure, similarly to bulk fluids. However, the modulus exceeds that of the bulk—the fluid in the pores appears much stiffer.

The effect of confinement in cylindrical pores is significantly greater than what is observed in slit pores. For a 3 nm pore in equilibrium with a reservoir pressure of 10 MPa, the bulk modulus of the confined fluid is more than doubled by the changed geometry. These results are explained by considering the environment observed by a fluid atom adsorbed close to the walls.^14^ When the pore surface is curved, the distance between the fluid atom and the rest of the wall is, on average, shorter than it would be if the wall were perfectly flat. An even greater effect would be observed in spherical pores, since the curvature is observed in three dimensions.^14^

### Bulk Modulus vs Inverse Pore Size

Finally, we apply the new method to investigate the fluid bulk modulus in pore sizes up to 100 nm in slit and cylindrical pores to demonstrate the gradual transition from confined to bulk-like behavior, illustrated in Figure 3. Corrente et al.^16^ showed the bulk modulus of methane in carbon slit pores of 2 to 9 nm is linear with respect to the inverse pore size below the critical pressure (4.6 MPa at 298 K). We have recaptured this effect, and confirm that similar behavior is observed in cylindrical pores. Even above the critical point, we observe largely linear behavior in smaller slit and cylindrical pores (less than 10 nm), with notable deviations from linearity only appearing toward the limit of a large pore. Overall, such behavior of a thermodynamic property of a fluid in a pore is similar to the Gibbs–Thomson effect, the change of the freezing/melting temperature in confinement.^17^

**Figure 3:**
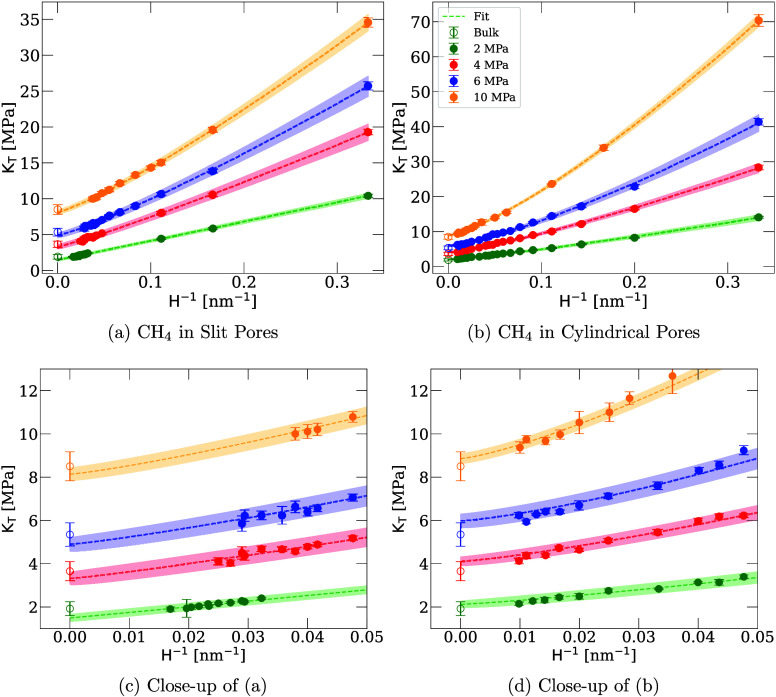
Bulk modulus of supercritical methane vs inverse pore size at reservoir pressures of 2 MPa – 10 MPa in slit pores (a) and (c), and cylindrical pores (b) and (d). The empty circles represent the bulk moduli of nonconfined methane at the corresponding reservoir pressure. The dashed lines are a curve fit to *K*_*T*_(*H* ^–1^; *a, b, c*) = *a*(*H* ^–1^)^*b*^ + *c*, and the shaded areas are the error of the fit.

Alongside the data in Figure 3, we plot corresponding best-fits for the function *K* = *aH* ^–*b*^ + *c* as dashed lines. This functional form captures the gradual approach toward the bulk limit while allowing expression of the effect of reservoir pressure, in which higher pressures, possible for shale gas formations,^35^ exhibit stronger nonlinear behavior than lower pressures. Specifically, the parameter *a* scales the increase in modulus over the bulk fluid and is directly related to the reservoir pressure. The parameter *b* controls the linearity of the trend, and generally increases with increasing reservoir pressure. The parameter *c* is the asymptote of the function, which directly represents the approximated modulus of the bulk fluid according on the trend of bulk modulus with increasing pore size. It is important to note that, although Figure 3 shows a slight underestimation of the fitted modulus for large slit pores, the predicted moduli from our new method agree with the moduli of bulk methane, as shown in Figure S4 of the Supporting Information.

## Discussion

When a fluid is confined in nanopores, its pressure varies in space according to the confining geometry and the distance from the pore walls. This means that the bulk modulus of a confined fluid is also spatially dependent. The fluid density near the solid wall is noticeably higher than the bulk density, and the local modulus of this high-density region is also much higher than the bulk modulus.^4,36,37^ However, when considering the mechanical response of a nanoporous solid saturated with fluid, probed via ultrasound, one generally observes the averaged properties of the fluid, and does not observe spatial variations on the scale of a single pore.^4^ It is not clear how the local bulk modulus at different coordinates within the pore volume might be experimentally probed. The works by Sun et al.^36,37^ showed that averaging this modulus over the pore space results in a value consistent with the thermodynamic approach for calculating the modulus used in this work. We focus accordingly on the average bulk modulus of the confined fluid.

The origin of the increased bulk modulus compared to the modulus in the bulk phase lies in the interaction between the pore walls and the fluid closely adsorbed to them. This interaction can be solvophilic (solid–fluid interaction is more favorable than fluid–fluid interaction, encouraging adsorption) or solvophobic (solid–fluid interaction is less favorable than fluid–fluid interaction, discouraging adsorption). It is the resultant structure of the fluid close to the walls which modifies the spatial average of the bulk modulus, the value probed by this simulation approach. The effect of the solid–fluid interactions on the bulk modulus has been studied previously.^38,39^

The reduction in computational cost afforded by the method demonstrated in this article varies widely depending on pore size, geometry, and fluid density. The use of integrated potentials significantly reduces the number of solid–fluid interactions that must be computed at each simulation step. When using explicit walls, fluid atoms interact with each solid atom in the nearby wall, whereas integrated potentials collapse those interactions into a single fluid-wall interaction. The proposed method also has a computational advantage over the MC method, owing to the parallelized MD simulations performed with LAMMPS and the lack of sampling inefficiencies that can hinder MC simulations (e.g., repeated attempts to insert new atoms in a dense fluid). Even so, this method does not completely eliminate the need for GCMC simulations, and in fact relies on GCMC to compute the average number of fluid particles in small pores. Furthermore, GCMC simulations remain an efficient tool for predicting fluid compressibility so long as the fluid is not too dense, the system is not too large, and the fluid–fluid interactions are not computationally expensive.

To compare GCMC simulations with MD simulations, it is helpful to use the metric of CPU-seconds per atom-step, which is the total number of real-world seconds needed to complete a simulation, multiplied by the number of CPU cores used, and divided by the product of the number of fluid particles and the total number of simulation steps. This metric normalizes performance so that simulations using different numbers of atoms and different simulation lengths can be quantitatively compared on equal footing. Our GCMC simulation in a 9 nm cylindrical pore using the integrated potential of eq 7 performed at an average speed of approximately 4.52 steps per second with an average of 636 methane particles, requiring 6.2 h on a single CPU core to acquire sufficient data (3.48 × 10^–4^ CPU-seconds per atom-step). For our MD simulation of the same pore size, simulating 157 particles was sufficient (the initial length of the pore being set to 3.6 nm). This simulation was performed with an average speed of over 1.6 × 10^4^ timesteps per second, parallelized across 4 CPU cores, requiring 35 min to acquire sufficient data (1.34 × 10^–6^ CPU-seconds per atom-step). This represents an improvement of more than 2 orders of magnitude.

## Conclusion

We have demonstrated a simulation technique that applies fluctuation theory to accurately model the bulk modulus of a confined Lennard-Jones fluid in a wide range of pore sizes. The parameters that define the interaction between the wall and fluid can easily be modified to represent other solid–fluid combinations. Carbon dioxide is one application of particular interest, such as in the adsorption of carbon dioxide in zeolites^40^ and other carbon capture utilization and sequestration (CCUS) operations. It will be necessary to specify the individual interactions between each constituent atom of the fluid and the walls (e.g., carbon and oxygen will have separate interaction parameters with the integrated potential). Furthermore, naturally occurring confined fluids are typically not pure, but mixtures, and there is an interest in studying competitive adsorption in nanopores.^41^ The proposed method is applicable to such systems, provided the fluid-wall interactions are also specified individually for each component. Care must also be taken when applying this method to polar fluids such as water. In very small pores, the hydrogen bonding network may cause the fluid to become heterogeneous and almost solid-like, which may adversely affect the measured volume fluctuations.

Integrated potentials can conceivably be applied for pore geometries other than idealized slits and cylinders, such as topologically rough slit pores and prisms with polygonal cross sections. Conceptually, the only requirement is the presence of at least one periodic dimension along which the fluid volume is allowed to fluctuate in a constant-stress ensemble. While this limitation prevents direct application to spherical pores, it may be possible to model them empirically by simulating slits and cylinders of various sizes and seeking to describe the combined effect of curvature and solid–fluid interactions on the fluid bulk modulus using the corresponding states principle for confined fluids.^3^ This method enables such a systematic approach, and is especially useful for complex fluids for which MC poses a hard limitation, such as flexible molecules and long alkanes.

## Supplementary Material





## Notes

Information about the integrated potentials for slit and cylindrical pores, details about the implementation of the integrated potentials and interaction parameters in LAMMPS, validation of the particle count extrapolation to large pores, estimation of statistical errors, fitting parameters for the bulk modulus as a function of the inverse pore size, and an analysis of the finite-size effects involved in calculating the bulk modulus from eq 4. (PDF)
LAMMPS source code for the integrated cylindrical potential, input files (LAMMPS format), and data files for Figures 2 and 3 and Figures S2–S4. (ZIP)
