# Supplementary material for: Compressibility of Confined Fluids from Volume Fluctuations
Source: Langmuir. 2025 Dec 15;41(51):34189–96. doi: 10.1021/acs.langmuir.5c03971 (PMC12756912; doi:10.1021/acs.langmuir.5c03971)
Supplement: Supplementary file 1 [file la5c03971_si_001.pdf]

Supporting Information: Compressibility of Confined Fluids from Volume Fluctuations

Jason Ogbebor,[†,¶] Santiago A. Flores Roman,[‡,¶] Geordy Jomon,[‡] and Gennady Y. Gor[*,‡]

†*Department of Materials Science and Engineering, Massachusetts Institute of Technology, Cambridge, Massachusetts, 02139, United States*

‡*Otto H. York Department of Chemical and Materials Engineering, New Jersey Institute of Technology, Newark, New Jersey, 07102, United States*

¶*These authors contributed equally to this work.*

E-mail: gor@njit.edu

LAMMPS Implementation

The integrated LJ potentials were implemented in the LAMMPS software, and are available on GitHub in the branch tjatjopoulos. Instructions for installing the potentials in LAMMPS are included in the input files of this work.

The exact syntax used to initialize the LJ walls representing slit pores was:

```
fix f_ID g_ID wall/lj104 zlo EDGE ${eps_sf} ${sig_sf} ${wall_cut} ${rho_A} ${nLayers}
${deltaLayers} zhi EDGE ${eps_sf} ${sig_sf} ${wall_cut} ${rho_A} ${nLayers}
```

`${deltaLayers}`, where `f_ID` is the fix ID, `g_ID` is the ID of the group of atoms interacting with the wall (fluid atoms), `${eps_sf}` and `${sig_sf}` are the fitted interaction parameters, `${wall_cut}` is the cutoff distance beyond which atoms do not interact with the wall, `${rho_A}` is the surface density of atoms in the pore wall, and `${nLayers}` and `${deltaLayers}` are the number of layers in the wall and the distance between those layers, respectively. The specification of a group ID other than `all` allows for mixtures to be simulated by overlaying walls on top of each other, each wall interacting with a different atom type through unique parameters. The walls take up the entire edge of the simulation box, even as the area of the edge changes during the NPT section of the procedure.

The exact syntax used to initialize the integrated potential representing cylindrical pores was:
`fix f_ID g_ID r_ID wall/region/tjatjopoulos ${eps_sf} ${sig_sf} ${rho_A} ${size}`, where `wall/region/tjatjopoulos` is the custom fix style and `${size}` is the diameter of the pore. `r_ID` is the region ID, which sets the cylindrical region of the pore. The region was defined as: `region r_ID cylinder x 0 0 $(size/2) INF INF side in`. This places the cylinder in the center of the box, with its central axis parallel to the x-axis. The cylinder is infinite in the axial direction so that the potential surface persists even as the length of the box in that direction increases.

TABLE S1: Lennard-Jones (LJ) interaction parameters for the simulations performed in this work. The "C - CH_4" parameters were calculated using the Lorentz-Berthelot mixing rules.[1] These parameters were used in Eq. S1 and Eq. S2.[2,3] The cut-off radius, r_{cut}, is the distance beyond which the LJ interactions are truncated and set to zero.

Interaction	ϵ (kcal/mol)	σ (nm)	r_{cut} (nm)
C - C	0.0556	0.340	-
CH_4 - CH_4	0.2941	0.373	1.20
C - CH_4	0.128	0.357	1.20

Potential by Tjatjopoulos et al.

The potential used to represent cylindrical pores was originally defined in Ref.[3] Its implementation in LAMMPS is based on the equations derived in Ref.[2] The ψ_n functions in the main text are defined as follows:

$$\psi_\mathrm{n}(r,R,\sigma) = 4\sqrt{\pi}\frac{\Gamma(n-\frac{1}{2})}{\Gamma(n)}\left(\frac{\sigma}{R}\right)^{2n-2}\left[1-\left(\frac{r}{R}\right)^2\right]^{2-2n} F\left[\frac{3-2n}{2},\frac{3-2n}{2};1;\left(\frac{r}{R}\right)^2\right] \quad \text{(S1)}$$

where Γ is the Gamma function, F is the hypergeometric function, and n is an integer or half-integer greater than one half.

Validation of Integrated Potential

To ensure that the integrated potential walls are accurate representations of a pore, the fluid density profiles across the height of the pore were compared. Two simulations of confined united-atom methane[4] at $T = 298\,\mathrm{K}$ were performed, once with all-atom graphitic walls and once with integrated potentials defined by:

$$U_\mathrm{sf} = \sum_{i=1}^{n}\left[4\pi\rho_\mathrm{s}\epsilon_\mathrm{sf}\sigma_\mathrm{sf}^2\left(\frac{1}{5}\left[\frac{\sigma_\mathrm{sf}}{r_i}\right]^{10}-\frac{1}{2}\left[\frac{\sigma_\mathrm{sf}}{r_i}\right]^{4}\right)\right], \quad \text{(S2)}$$

as described in the main text. See Table S1 for the LJ parameters governing methane-methane and methane-wall interactions. Both simulations used the same pore area (4.272 nm $\times$ 4.932 nm as in Ref.[5]) and pore size (3 nm). Figure S1 compares the resultant density profiles of the confined fluid. The agreement between density profiles gives assurance that the fluid in an implicit pore (represented by an external potential) should exhibit the same physical behavior as it would exhibit in an explicit, all-atom pore.

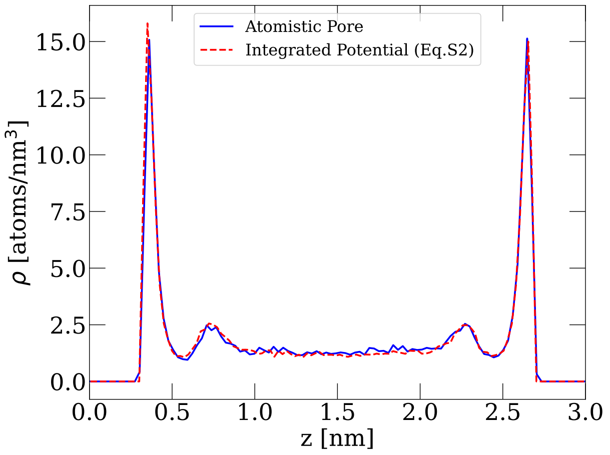

(a)

FIGURE S1: Density profiles of methane in a 3 nm carbon slit pore. The blue line represents the fluid density in a physical, atomistic pore. The red dashed line represents the fluid confined between integrated potential walls defined by Eq. S2

Extrapolation of Particle Count in Large Pores

The method proposed in the main text requires an initial density to populate the simulation box. One approach to obtain the initial density is from GCMC simulations. As long as we know $\mu = \mu(P)$ (for example, from an equation of state), we can predict the density of the fluid in a pore using GCMC simulations and then compute the bulk modulus from MD simulations. However, simulating large systems using GCMC can be lengthy. To avoid running additional GCMC simulations, we propose predicting the density of the fluid confined in small pores using GCMC simulations and then extrapolating it to large pores. The proof is explained in the following.

We reproduced the adsorption of methane in cylindrical and slit pores (sizes ranging from 3 nm to 50 nm) using GCMC simulations at 298 K and pressures ranging from 2 MPa to 10 MPa. The simulations were run for 10^6 MC equilibration steps and 10^8 production steps. The chemical potential of bulk methane was set according to CoolProp under the defined conditions.[6] Solid-fluid and fluid-fluid interactions were the same as those of the MD simulations, and the effective surface density was set to $\rho_s = 38.19\,\text{nm}^{-2}$ for both cylindrical and slit pores.[2]

The excess adsorbed amount is defined as $N_{\mathrm{ex}} = (\rho_{\mathrm{f}} - \rho_{\mathrm{b}})V_{\mathrm{f}}$, where ρ_{f} is the density of confined methane and ρ_{b} is the density of bulk methane. V_{f} is the fluid-accessible volume in the pore. The predicted N_{ex} from GCMC simulations was then fitted according to:

$$N_{\mathrm{ex}}(V_{\mathrm{f}}; B, \alpha, \theta) = B\frac{V_{\mathrm{f}}^{\alpha-1}e^{-V_{\mathrm{f}}/\theta}}{\theta^{\alpha}\Gamma(\alpha)}, \tag{S3}$$

where B, α, and θ are the fitting parameters. The resulting density of the confined fluid is:

$$\rho_{\mathrm{f}} = \rho_{\mathrm{b}} + N_{\mathrm{ex}}/V_{\mathrm{f}}. \tag{S4}$$

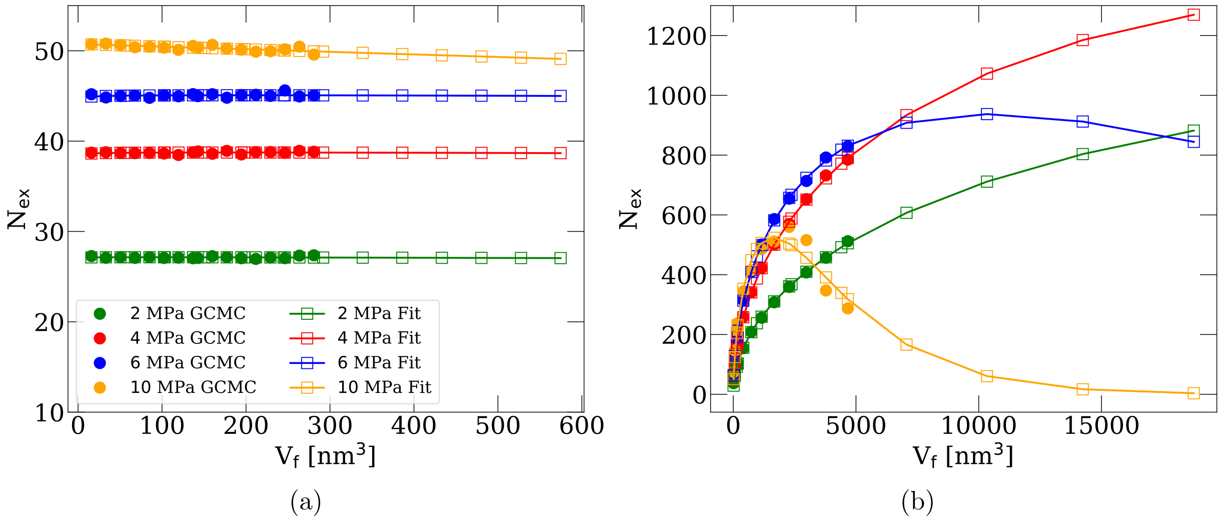

FIGURE S2: Predicted (filled circles) and estimated (empty squares, after fitting Eq. S3) excess adsorbed amount N_{ex} of methane in (a) slit pores and (b) cylindrical pores at pressures ranging from 2 MPa to 10 MPa and 298 K as a function of fluid volume in the pore, V_{f}. Simulated pores ranged from 3 nm to 50 nm. Excess adsorbed amount was estimated from 3 nm to 100 nm.

Figure S2 shows the predicted and excess adsorbed amount for slit and cylindrical pores. According to GCMC predictions, N_{ex} strongly depends on the pore shape. For slit pores, N_{ex} is mostly constant as a function of pore size. However, for cylindrical pores, N_{ex} reaches a maximum that depends on the thermodynamic conditions, i.e., the maximum shifts to lower pore sizes as the pressure increases. The Gamma distribution can be easily adjusted according to the pore shape by the shape parameter α. The resulting values were $\alpha \approx 1$ for

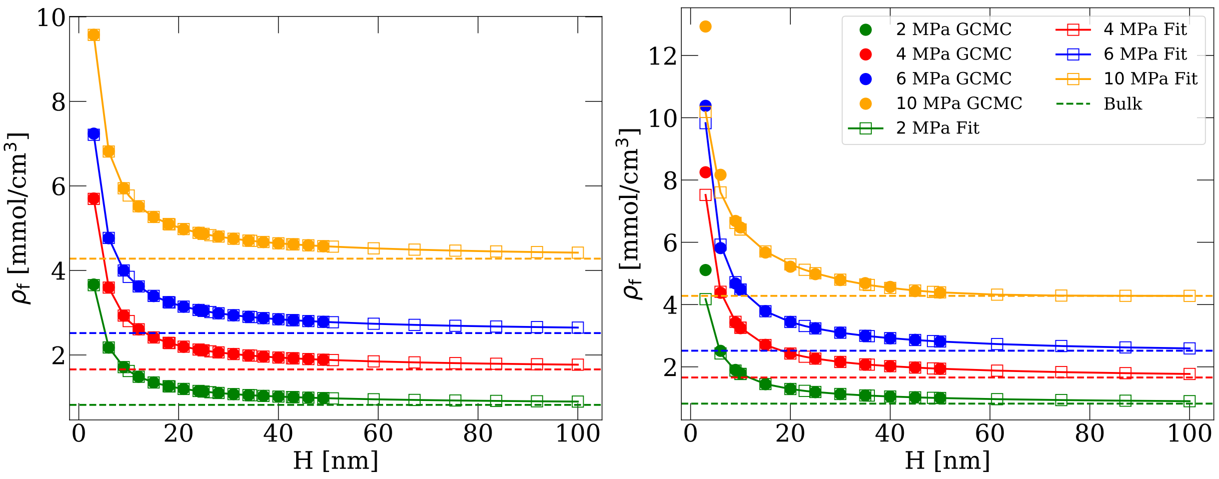

(a) (b)

FIGURE S3: Predicted (filled circles) and estimated (empty squares, calculated from Eq. S4) fluid densities ρ_f of methane in (a) slit pores and (b) cylindrical pores at pressures ranging from 2 MPa to 10 MPa and 298 K as a function of pore size. Simulated pores ranged from 3 nm to 50 nm. Densities were estimated from 3 nm to 100 nm. Dashed lines are the bulk densities of methane.

slits and $\alpha \approx 1.5$ for cylinders.

Once we have an estimate of the fluid density from Eq. S4, we can extrapolate it to a pore of 100 nm in size. Figure S3 shows the predicted and estimated densities of methane in cylindrical and slit pores from 3 nm to 100 nm. The estimated densities closely match predictions from GCMC simulations.

It is important to note that, for slit pores, Eq. S3 succeeds in estimating N_{ex}. However, for cylindrical pores, N_{ex} is underestimated at low pore sizes. However, as the pores become larger, ρ_b in Eq. S4 becomes dominant in determining ρ_f. Since our study focuses on large mesopores ($H \geq 10$ nm), we can disregard the mismatches at $H < 10$ nm.

Estimating Statistical Errors of Predicted Bulk Moduli

We estimated the statistical error of K_T through the bootstrap method.[7] This method takes n random subsets of size n (with replacement) of the collected data (the computed volume at every given timestep, or the computed number of molecules at every given MC step) to recompute the statistic n times. Here, our statistic is the bulk modulus. If n is large enough

($n > 100$), then the n resamples will form a bootstrap distribution of the statistic with an estimated error:

$$\varepsilon[K_T] = \sqrt{\langle K_T^2 \rangle - \langle K_T \rangle^2}. \tag{S5}$$

Moreover, from the distribution, we can choose our confidence interval. In this work, we resampled $n = 500$ subsets from datasets of 10^4 values to estimate the error of K_T with 95% confidence.

Fitting Parameters for Bulk Modulus Dependence on Inverse Pore Size

The error of the fit of $K_T = K_T(H^{-1})$ was estimated as

$$\varepsilon[K_T] = \sqrt{(H^{-1})^{2b}(\varepsilon[a])^2 + ab(H^{-1})^{2(b-1)}(\varepsilon[b])^2 + (\varepsilon[c])^2}, \tag{S6}$$

where $(\varepsilon[a])^2$, $(\varepsilon[b])^2$, and $(\varepsilon[c])^2$ are the corresponding variances of the fitting parameters a, b and c.

Table S2: Results from fitting the bulk modulus vs pore size to the function $K_T(H) = aH^{-b} + c$ using data from cylindrical pores. Note that the parameter c represents the bulk modulus of the non-confined fluid, K_{Bulk}. K_{Bulk} was not included in the fitting data. R^2 is the coefficient of determination. The errors of the fitting parameters are their variances (errors lower than 10^{-3} units were excluded).

$P_{\text{reservoir}}$ (MPa)	a (nm · MPa)	b (unitless)	c (MPa)	K_{Bulk} (MPa)	R^2
2	43.16 ± 1.35	1.17 ± 0.02	2.07 ± 0.06	1.92 ± 0.32	0.9982
4	94.37 ± 2.43	1.24 ± 0.02	4.06 ± 0.09	3.65 ± 0.44	0.9987
6	148.92 ± 4.90	1.32 ± 0.03	5.95 ± 0.16	5.35 ± 0.54	0.9983
10	259.30 ± 4.17	1.31 ± 0.01	8.89 ± 0.12	8.50 ± 0.67	0.9998

TABLE S3: Results from fitting the bulk modulus vs pore size to the function $K_T(H) = aH^{-b} + c$ using data from slit pores. Note that the parameter c represents the bulk modulus of the non-confined fluid, K_{Bulk}. K_{Bulk} was not included in the fitting data. R^2 is the coefficient of determination. The errors of the fitting parameters are their variances (errors lower than 10^{-3} units were excluded).

$P_{\text{reservoir}}$ (MPa)	a (nm · MPa)	b (unitless)	c (MPa)	K_{Bulk} (MPa)	R^2
2	27.13 ± 0.18	1.01	1.49	1.92 ± 0.32	0.9998
4	54.52 ± 3.2	1.12	3.32 ± 0.02	3.65 ± 0.44	0.9992
6	75.42 ± 5.63	1.17	4.89	5.35 ± 0.54	0.9991
10	98.52 ± 4.04	1.20	8.14 ± 0.02	8.50 ± 0.67	0.9997

Finite-Size Dependence of the Bulk Modulus from Volume Fluctuations

Finite-size effects may arise due to the dependence of K_T on volume fluctuations. Hence, it is essential to analyze the dependence of K_T on the box size along the periodic directions. Fig. S4 shows the resulting K_T of methane in a 59 nm slit pore as a function of the wall length in the range $3r_{\text{cut}} \leq L_{\text{x}} \leq H$, where $r_{\text{cut}} = 1.2$ nm. Note that L_{x} comes from $A = L_{\text{x}} \times L_{\text{x}}$, where A is the surface area of a wall. For a pore size of $H = 59$ nm, it is expected to have K_T approaching the modulus of its bulk phase, K_{Bulk}. However, for sufficiently small wall lengths (in this case, $L_{\text{x}} \lesssim 16$ nm), the predicted K_T underestimates K_{Bulk}, while for large enough wall lengths ($L_{\text{x}} \gtrsim 16$ nm), our predictions fall around K_{Bulk}. To predict fluid thermal properties, we must widely cover the phase space, so we can obtain a good estimate of the variance ($\langle \delta V \rangle^2$ for our method). If our system is small in the amount of molecules or highly dense, we may not be able to sufficiently sample the phase space, resulting in underestimated predictions. For $L_{\text{x}} = 16$ nm, we simulated ~ 8560 methane molecules. Although this effect has been observed mostly on MD predictions of transport properties, the dependence of our method on barostat protocols makes K_T potentially sensitive to finite-size effects.[8] Finite-size effects may also depend on the pore geometry. Unlike slit pores, we did not observe any such dependence in cylindrical pores for wall lengths $L_{\text{x}} \geq 3r_{\text{cut}}$.

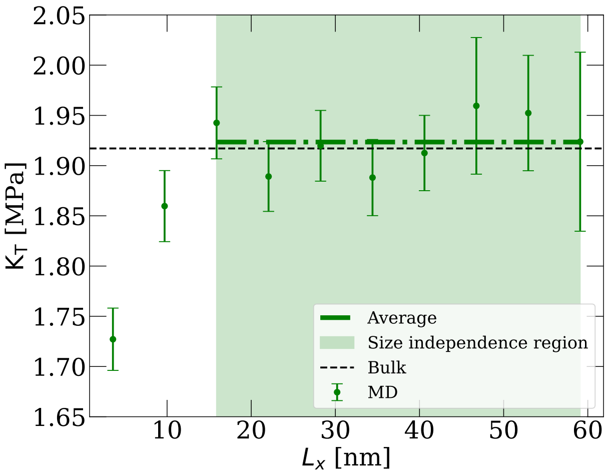

(a)

FIGURE S4: Predicted bulk modulus of methane in a 59 nm slit pore at 2 MPa and 298 K as a function of the wall length, L_{x}. The dashed line is the modulus of the bulk phase at the same thermodynamic conditions. The thick dashed-dotted line is the average of K_T in the size-independent region ($L_{\mathrm{x}} \gtrsim 16$ nm).

Gaussian Distribution Criterion for Volume Fluctuations

We can ensure that the equation

$$K_T = \frac{k_{\mathrm{B}} T \langle V \rangle}{\langle \delta V^2 \rangle} \tag{S7}$$

predicts the bulk modulus, K_T, if we assume that the fluid volume fluctuates according to a Gaussian distribution. The reason is as follows.

Let us assume that there is a sufficient number of particles in the simulation box (larger than 50), and that the fluid is homogeneous. At sufficiently high temperatures (quantum effects are negligible), the probability w of a fluctuation is proportional to the change of total entropy in a closed system, $w \propto \exp(\delta S_{\mathrm{tot}})$. We can equally relate δS_{tot} to the minimum work needed to carry out fluctuations in thermodynamic quantities, i.e.,

$$w \propto \exp\left(-\frac{\delta P \delta V - \delta T \delta S}{2T}\right), \tag{S8}$$

where δP, and δT are pressure and temperature fluctuations, respectively. The Boltzmann factor k_{B} is implicit in the temperature variable T. If we take V and T as independent

variables, then we can derive

$$w \propto \exp\left[-\frac{C_v}{2T^2}\left(\delta T\right)^2 + \frac{1}{2T}\left(\frac{\partial P}{\partial V}\right)_T \left(\delta V\right)^2\right], \tag{S9}$$

where C_v is the heat capacity at constant volume. Then, we can split the exponential term of Eq. S9 into a product of exponentials. This implies that temperature and volume fluctuations are statistically independent. Focusing on the volume-dependent term in the exponential of Eq. S9, we can relate the volume fluctuations to a Gaussian distribution, hence

$$\langle(\delta V)^2\rangle = -T\left(\frac{\partial V}{\partial P}\right)_T. \tag{S10}$$

Therefore, from the definition of the isothermal bulk modulus, we obtain

$$K_T \equiv -V\left(\frac{\partial P}{\partial V}\right)_T = \frac{TV}{\langle(\delta V)^2\rangle}, \tag{S11}$$

where V and $\langle(\delta V)^2\rangle$ are the mean and variance of fluctuating volume. The procedure is explained in more detail by Landau and Lifshitz.[9]

Even though we assumed a homogeneous fluid, Eq. S7 is still applicable to confined fluids if we only treat the periodic directions where the fluid still behaves as homogeneous.

Since the Gaussian distribution is a necessary condition to make Eq. S7 applicable to our system, we computed the volume histogram for each of our MD simulations in the NPT ensemble. Figure S5 shows the volume density histograms of methane in 6 nm slit and cylindrical pores at 10 MPa. For a sufficiently large number of samples (more than 10^4), our histograms approach Gaussian distributions. Therefore, we can use Eq. S7 to effectively predict the bulk modulus of methane in both slit and cylindrical pores.

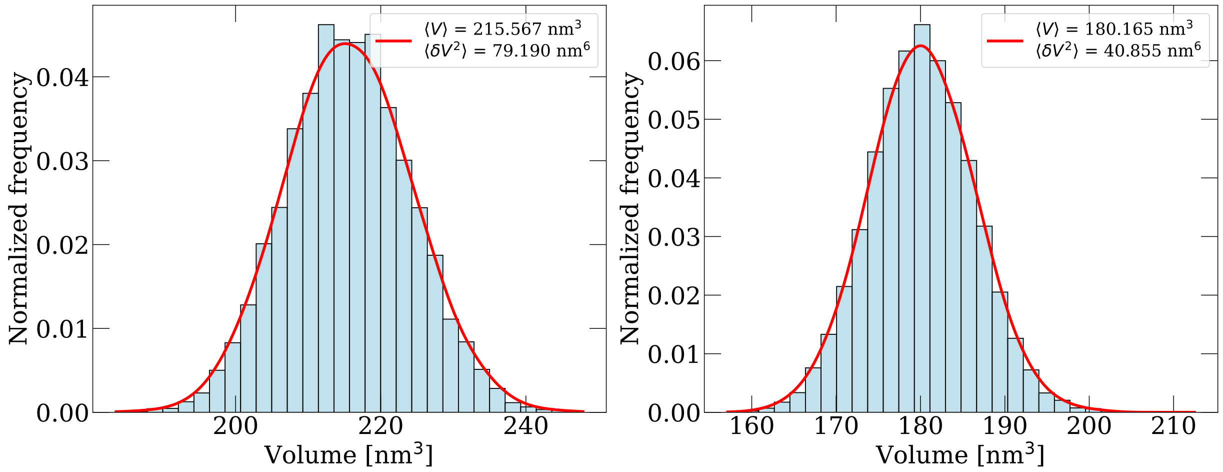

(a) (b)

Figure S5: Volume histograms of methane in 6 nm slit (a) and cylindrical (b) pores at 10 MPa. We collected 2×10^4 values to reproduce a Gaussian-shaped distribution.

References

(1) Lorentz, H. A. Ueber die Anwendung des Satzes vom virial in der Kinetischen Theorie der Gase. *Annalen der Physik* **1881**, *248*, 127–136.

(2) Siderius, D. W.; Gelb, L. D. Extension of the Steele 10-4-3 potential for adsorption calculations in cylindrical, spherical, and other pore geometries. *J. Chem. Phys.* **2011**, *135*, 084703.

(3) Tjatjopoulos, G. J.; Feke, D. L.; Mann Jr, J. A. Molecule-micropore interaction potentials. *J. Phys. Chem.* **1988**, *92*, 4006–4007.

(4) Martin, M. G.; Siepmann, J. I. Transferable potentials for phase equilibria. 1. United-atom description of n-alkanes. *J. Phys. Chem. B* **1998**, *102*, 2569–2577.

(5) Corrente, N. J.; Dobrzanski, C. D.; Gor, G. Y. Compressibility of Supercritical Methane in Nanopores: A Molecular Simulation Study. *Energy Fuels* **2020**, *34*, 1506–1513.

(6) Bell, I. H.; Wronski, J.; Quoilin, S.; Lemort, V. Pure and Pseudo-pure Fluid Thermo-

physical Property Evaluation and the Open-Source Thermophysical Property Library CoolProp. *Ind. Eng. Chem. Res.* **2014**, *53*, 2498–2508.

(7) Newman, M. E. J.; Barkema, G. T. *Monte Carlo Methods in Statistical Physics*; Oxford University Press, 1999; Chapter 3.4.3.

(8) Kim, K.-S.; Han, M. H.; Kim, C.; Li, Z.; Karniadakis, G. E.; Lee, E. K. Nature of Intrinsic Uncertainties in Equilibrium Molecular Dynamics Estimation of Shear Viscosity for Simple and Complex Fluids. *J. Chem. Phys.* **2018**, *149*, 044510.

(9) Landau, L. D.; Lifshitz, E. M. *Statistical Physics, vol. 5*; Pergamon, 1980; Vol. 30; Chapter 112.
